# Acute Effects of Beetroot Juice Supplements on Resistance Training: A Randomized Double-Blind Crossover

**DOI:** 10.3390/nu12071912

**Published:** 2020-06-28

**Authors:** Antonio Ranchal-Sanchez, Victor Manuel Diaz-Bernier, Candelaria Alonso De La Florida-Villagran, Francisco Jesus Llorente-Cantarero, Julian Campos-Perez, Jose Manuel Jurado-Castro

**Affiliations:** 1 Maimonides Biomedical Research Institute of Cordoba (IMIBIC), University of Cordoba, 14004 Córdoba, Spain; en1rasaa@uco.es; 2 Department of Nursing, Pharmacology and Physiotherapy, Faculty of Medicine and Nursing, University of Cordoba, 14071 Córdoba, Spain; victormd2007@gmail.com (V.M.D.-B.); candelalonso11@gmail.com (C.A.D.L.F.-V.); 3 Department of Specific Didactics, Faculty of Education, University of Cordoba, 14071 Córdoba, Spain; llorentefj@yahoo.es; 4 CIBEROBN, (Physiopathology of Obesity and Nutrition) Institute of Health Carlos III (ISCIII), 28029 Madrid, Spain; 5 Department of Food Science and Technology, Rabanales University Campus, University of Cordoba, 14071 Córdoba, Spain; m02capej@uco.es; 6 Metabolism and Investigation Unit, Maimonides Biomedical Research Institute of Cordoba (IMIBIC), Reina Sofia University Hospital, University of Cordoba, 14004 Córdoba, Spain

**Keywords:** beet juice, dietary supplement, nitrate, nitric oxide, ergogenic aid, athletic performance

## Abstract

The ingestion of beetroot juice (BJ) has been associated with improvements in physical performance in endurance sports, however the literature on resistance training (RT) is scarce. The aim of this study was to investigate the acute effects of BJ compared to a placebo (PLA) on muscular endurance and movement concentric velocity during RT. Twelve healthy men performed an incremental RT test (back squat and bench press) with three sets, at 60%, 70%, and 80% of their repetition maximum (1-RM). Movement velocity variables, total number of repetitions performed until concentric failure, blood lactate, and ratings of perceived effort post-training were measured. A higher number of repetitions were recorded with BJ compared to those with PLA (13.8 ± 14.4; *p* < 0.01; effect size (ES) = 0.6). Differences were found at 60% 1-RM (9 ± 10; *p* < 0.05; ES = 0.61) and 70% 1-RM (3.1 ± 4.8; *p* < 0.05; ES = 0.49), however, no differences were found at 80% 1-RM (1.7 ± 1; *p* = 0.12; ES = 0.41). A greater number of repetitions was performed in back squat (13.4 ± 13; *p* < 0.01; ES = 0.77), but no differences were observed in bench press (0.4 ± 5.1; *p* = 0.785; ES = 0.03). No differences were found for the rest of the variables (*p* > 0.05). Acute supplementation of BJ improved muscular endurance performance in RT.

## 1. Introduction

Sports supplements based on beetroot juice (BJ) have been studied recently, with a great deal of interest shown by the field of sports nutrition, as well as sports performance [1], and a focus on the effects on reducing cardiovascular diseases and coronary heart disease [1,2]. The principal active principle of BJ supplementation is based on its levels of dietary inorganic nitrate (NO_3_^−^). After ingestion, the NO_3_^−^ present in BJ enters the whole salivary circulation, where it is absorbed and reduced to nitrite (NO_2_). This process is due to the action of nitrate reductase facultative anaerobic bacteria present at the dorsal surface of the tongue [3]. This causes an increase in NO_2_ levels in the stomach, where NO_2_ is decomposed into nitric oxide (NO) and gets into the systemic circulation [4,5].

Regarding these metabolic pathways, the NO precursors mainly cause a vasodilator effect, achieving a dilation of the vascular endothelium [6,7]. These alterations could have potential benefits in the sport performance by increasing muscle blood flow [8], altering and improving lactate removal [9], and lowering blood pressure [10]. In addition, it has been shown that the intake of NO_3_^−^ produces an enhancement of economy, reducing oxygen consumption (VO_2_) during exercise [1], which may indicate an improvement in adenosine triphosphate (ATP) synthesis due to a reduction of VO_2_ cost [11]. Thanks to these benefits of BJ supplements, BJ consumption and its effects have been widely evaluated and constated in endurance sports as athletics, cycling, time trials, and rowing, where the cardiovascular system has an important role for sport performance [1,6,12].

However, BJ supplements in resistance training (RT) [13,14,15] have received less attention [16]. Mosher et al. [14] supplemented 12 active men for six days with BJ and placebo (PLA), observing an increase in muscular endurance in terms of weightlifting and number of repetitions. Similarly, Bailey et al. [13] recruited seven active men who consumed BJ for six days, reporting an improvement of strength, reducing the total use of ATP and increasing the concentration of NO_3_ in the plasma. In addition, previous studies have shown that BJ preferentially increases blood flow [8] and muscle force contraction [17] in muscle fiber type II, but not type I. Therefore, it is possible that BJ could enhance performance in a type of exercise that recruits type II fibers, e.g., RT. Additionally, Flanagan et al. [18] observed an improvement after 3 days of supplementation on an NO_3_^−^ rich bar on the optimal rate of motor units and in the ability to sustain the electromyography (EMG) peak of amplitude over the course of a strenuous heavy resistance exercise protocol; however, they did not find improved performance on an RT protocol designed to assess neuromuscular fatigue. Collectively, these findings suggested that an NO_3_^−^ rich supplement could enhance neuromuscular efficiency during fatiguing resistance exercise.

RT athletes should prioritize movement velocity in their workouts, velocity-based RT, and especially high-velocity training, has greater advantages for the improvement of sport performance than traditional training [19]. To date, only a recent study [15] has investigated the effect of an acute dose on RT in bench press exercise measured by movement velocity, with a load protocol typically used to induce hypertrophy, 67–85% of the one repetition maximum (1-RM) [20,21], indicating that acute BJ supplementation positively impacts velocity, power, and total repetitions during free-weight bench press exercise. However, the acute effects of BJ on squat exercise, or the combination of more than one strength exercise, have not been studied.

Thus, due to the possible benefits of the BJ supplementation in the sport performance and to the limited investigation about BJ supplementation in RT and velocity-based RT, the purpose of this study was to investigate the possible ergogenic effect of BJ on: (i) the movement velocity and power produced in the concentric phase during resistance exercise; (ii) muscular endurance measures such as the maximal number of repetitions until concentric failure. In addition, we also observed physiological effects, with the measurement of lactate and ratings of perceived exertion (RPE) by the participants.

## 2. Material and Methods

### 2.1. Participants

Twelve healthy adult men aged between 20 and 28 who practiced recreational RT were recruited, with at least 2 years of experience in RT, familiarized with back squat and bench press exercise. None of the participants were competitive athletes. All participants included in the study were moderately physically active during the 6 months before the study (RT sessions of at least three days a week). Participants were familiarized with performing RT to failure, which was included in their training routines. Not meeting at least these characteristics was considered a reason for exclusion. Table 1 shows the characteristics of the participants.

**Figure 1:**
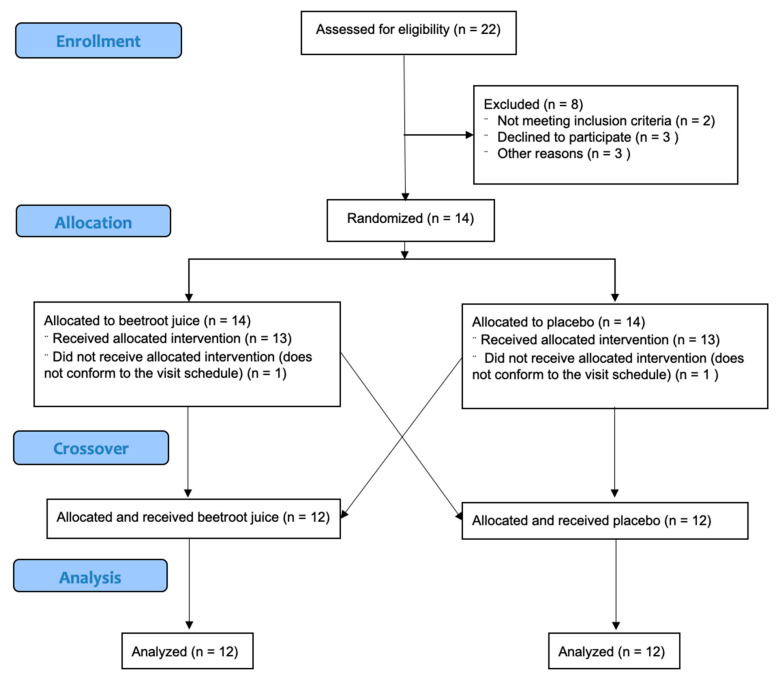
CONSORT diagram.

Individuals with low blood pressure and any musculoskeletal injuries that could prevent them following the exercise protocol were excluded. Any individuals who consumed any type of nutritional substance, supplement, or anabolic substances in the three months prior to or during the study were also excluded. These inclusion criteria were verified by personal interviews. Those participants who did not complete all stages of the experimental protocol were also excluded. Figure 1 presents a CONSORT diagram.

The participants recruited were briefed on the protocol and objective of the study, and they signed a written consent prior to the start of the research. The study was conducted in accordance with the Declaration of Helsinki, and the protocol was approved by the Ethics Committee of the Andalusian Ethics Portal of Biomedical Research (protocol code: BEETROOT JUICE; record nº287).

### 2.2. Design

The study involved an experimental double-blind crossover randomized trial. The experimental procedure consisted of three visits, separated from each other by 72 h, to achieve the total elimination of any effects caused by the BJ [22], as well as the optimal recovery of the participants [23]. The experimental test measurements were taken between 10 a.m. and 2 p.m., at the same time of day (±0.5 h) for each individual, to standardize the influence of the circadian rhythm, at a temperature of 24 °C (±1 °C). During the first visit, the participants’ height (Seca 214 portable stadiometer; Seca, Hamburg, Germany) and body composition measured by bioimpedance (Tanita MC-780MA; Tanita Corporation, Japan) were recorded. The manual pressure force (Dynamometer TKK 5101; Takei, Tokyo, Japan) was also determined. In addition, a technique familiarization of the exercises and materials was performed. It was defined as the maximum load the individual was able to lift with the appropriate exercise action. Later, the participants returned to the laboratory a further two times to perform an incremental resistance training test.

### 2.3. Familiarization Protocol and One-Repetition Maximum Testing

The participants performed a familiarization with the lifting protocol with the maximum explosive velocity in back squat and bench press. The participants lifted 20 kg on a Smith machine as quickly and explosively as possible for a total of three repetitions and were given form correction if necessary. Before starting the protocol, to know the 1-RM, a warm-up based on previous studies that studied the effects of BJ on RT [14,15] was carried out, checking that the participants could bear it well. Testing protocol to find 1-RM load in back squat and bench press followed the suggestions proposed by Brown and Weir [24]. Both exercises were performed on the same day and the protocol was replicated for back squat and bench press. During the first set, subjects performed five repetitions with 50% of the estimated 1-RM. In the second set, they performed three repetitions with 70% of the estimated 1-RM, with 3 min intervals between them. After the second set, subjects rested for 3 min. Then, the athletes had up to five trials to achieve the 1-RM load, with a 3 min interval between trials. Because the movement velocity was recorded during the protocol, it was verified that the velocity of the 1-RM load matched the 1-RM speed described for each exercise [25,26]. This protocol was used to determine the intensity percentages of the exercise in later stages.

### 2.4. Supplement Protocol

BJ and PLA were poured into opaque jars, prepared by a food technologist (C.A.D.L.F-V.), together with a third person from outside the research team. The supplements were prepared in a laboratory in order to guarantee the entire process of food safety, moreover, adequate transport and storage conditions for the product were ensured. The order in which the participants consumed one supplement or the other was randomly and double-blindly assigned by the third person outside the research team, who was responsible for encoding the jars. The order in which the BJ or PLA was consumed was only known by the researchers when all the data had been collected.

One of the drinks, 70 mL of BJ (BEET It Sport^®^; James White Drinks Ltd., Ipswich, UK) or 70 mL of blackcurrant beverage, which depleted NO_3_^−^ unlike BJ as a PLA (Capri-Sun, Uxbridge, UK), was taken 120 min before each visit [1]. Participants completed a 24 h dietary recall, on the day before to the first visit, as a way to check that participants replicated their diet each day of the exercise trials, as suggested in previous work [15]. To avoid a possible confusing influence of the usual intake of NO_3_^−^ in the diet, participants received a list of foods rich in NO_3_^−^ (e.g., beetroot, celery, or spinach) to avoid for 48 h before each session. 

All the participants were instructed: (i) to eliminate ingestion on foods rich in NO_3_^−^, stimulants (e.g., caffeine), gum or sweets, or alcohol that could alter the oral microbiota during the three days before the laboratory visit; (ii) to avoid brushing their teeth on the morning of testing and not to use antiseptic rinses from one week prior to the first laboratory visit and for the duration of the study, due to a potential prevention of the desired increase in nitrite levels after ingestion of NO_3_^−^ [27]; (iii) the participants guaranteed an adequate level of hydration; (iv) to avoid strenuous exercise during the research period; (v) and to sleep for at least 8 h.

The BJ supplement used contained 6.4 mmol·L^−1^ or 400 mg NO_3_^−^ per serving: the content claimed by the manufacturer had been previously validated in a study to verify the nutritional veracity of various BJ supplements, this quantity of NO_3_^−^ is sufficient to generate an ergogenic effect [28]. 

Since the participants were aware of the study hypothesis, they were asked whether they had previously consumed BJ (i.e., “Have you ever consumed BJ?”). In addition, once they completed each of the visits, they were asked whether they recognized the type of supplement they consumed (i.e., “Which supplement do you think you have ingested? (a) BJ; (b) PLA; (c) Don’t know”).

### 2.5. Exercise Protocol and Movement Velocity Measurement

At each visit, the participants completed a supervised warm-up in which they performed 8 min of gentle muscle activation in a cycle ergometer (Ergomedic 828E, Vansbro, Sweden), followed by 12 repetitions of back squat with a determined load of 10% 1-RM. After a rest period of 1 min, they performed six repetitions, increasing the load to 30% 1-RM. They were allowed 1 min of rest and the same protocol was followed on the bench press. A similar warm-up was proposed in comparable previous researches [14,15]. Once completed, 2 min passed before the start of the incremental strength test. 

The test, firstly back squat followed by bench press, with a 3 min rest between the two activities, was divided into three series with load volumes of 60%, 70%, and 80% 1-RM, respectively, and performing repetitions until muscle failure. These were carried out incrementally and with a 3 min rest between each series [29], allowing muscle fatigue to be recovered and the maximum number of repetitions reached in each series. The execution velocity was controlled by the researchers using a recently validated linear position transducer (v.4.1, Speed4Lift; Madrid, Spain), with a coefficient of variation (2.61%) with respect to the “gold-standard” (V120:Trio; OptiTrack, NaturalPoint, Inc.), and compared with seven devices for measuring the execution velocity [30]. The participants were motivated to perform at their maximum velocity in the concentric phase of each repetition to ensure the use of maximum muscle strength [25]. The complete test lasted approximately 35 min (including warm-up time), which was previously estimated in a protocol training session conducted by the researchers together with trained volunteers; these volunteers were not included as study participants. This estimate was later corroborated during the study (33 ± 5 min).

In order to guarantee complete vertical linearity and minimize the velocity measurement variability in each repetition, a Smith machine (Technogym, Barcelona, Spain) was used, so that the participant paused briefly after the eccentric phase (between 1 and 1.5 s), supported by either the bar in a support, or the chest. This limited the countermovement, enabling greater control and reproducibility of the measurement in the concentric phase of movement [31]. For back squat execution, the subject was placed with the feet apart at shoulder height and the bar was placed on the shoulder blades with the hands gripping the bar, and then they flexed the knees at 120° followed by its extension to the original standing position. For bench press, repetitions were completed throughout a full range of motion in elbow flexion–extension [32].

During the study, the load in kg lifted and the maximum number of repetitions at 60–70–80% 1-RM until failure (back squat and bench press) were recorded, in addition to, the mean propulsive velocity in the concentric movement phase of each repetition, in order to obtain the maximum velocity (MV), and maximum power (MP) in Watt (W) in each set of different RM percentages. Measurements obtained were exported from the device software. The highest value of the first two repetitions was extracted from the MV and MP measurements. These values were used for further analysis.

### 2.6. Lactate Measurement and Ratings of Perceived Exertion

Lactate was objectively determined in blood using the Lactate Scout + (EKF Diagnostics, Leipzig, Germany), and collecting the samples 3 min after the exercise test for both conditions (BJ or PLA) [33]. Samples were obtained from the ear lobe, a conventional sampling site [34], after the lobe was cleansed and sterilized with 70% ethanol. Additionally, we obtained RPE values on a scale of 1–10 [35]. Specifically, subjects were instructed to report their perceived exertion immediately at the end of the protocol test. They were told that numerical value 2 correspond with easy, 3–4 somewhat easy, 6 somewhat hard, 8–9 hard, and 10 extremely hard.

### 2.7. Statistical Analysis

To study the normality of the variables, Shapiro–Wilk tests were performed. A two-way ANOVA with repeated measures was applied for the effect of time (different sets analyzed: 60%, 70%, and 80% 1-RM), condition (BJ vs. PLA), and the time × condition interaction on the total number of repetitions performed, velocity, and power. The Greenhouse–Geisser adjustment for sphericity was calculated. After a significant F-test, differences among means were identified using pairwise comparisons with Bonferroni’s adjustment. The comparison of the mean (repetitions to failure, velocity, power, lactate, and RPE) outcomes between the two experimental conditions (BJ and PLA) was realized with a paired-samples t-test. Effect size (ES) was calculated following the method set out by Lakens [36] using Cohen’s *d* as the ES index, where ES could be roughly classified as small (<0.2), medium (0.5 to 0.8), and large (>0.8) [37]. The data are displayed as the mean difference (MD) ± their standard deviation (SD), or comparing the mean ± SD for each condition, continued from ES. Significance was set at *p* < 0.05 or *p* < 0.01. The sample size was chosen based on recent work on the effects of BJ on strength training [14,15]. In a similar population, and using the calculation based on a normal distribution, a sample was selected that should have at least 11 individuals attending an estimated proportion of 95% with an accepted error (or precision) of 5% and a confidence level of 95%. SPSS software v22 (IBM, Portsmouth, United Kingdom) was used for the statistical analysis.

## 3. Results

All participants had not previously consumed BJ, therefore they did not know its flavor. However, 66% of the sample correctly identified the BJ supplementation (Supplementary Table S1).

**Figure 2:**
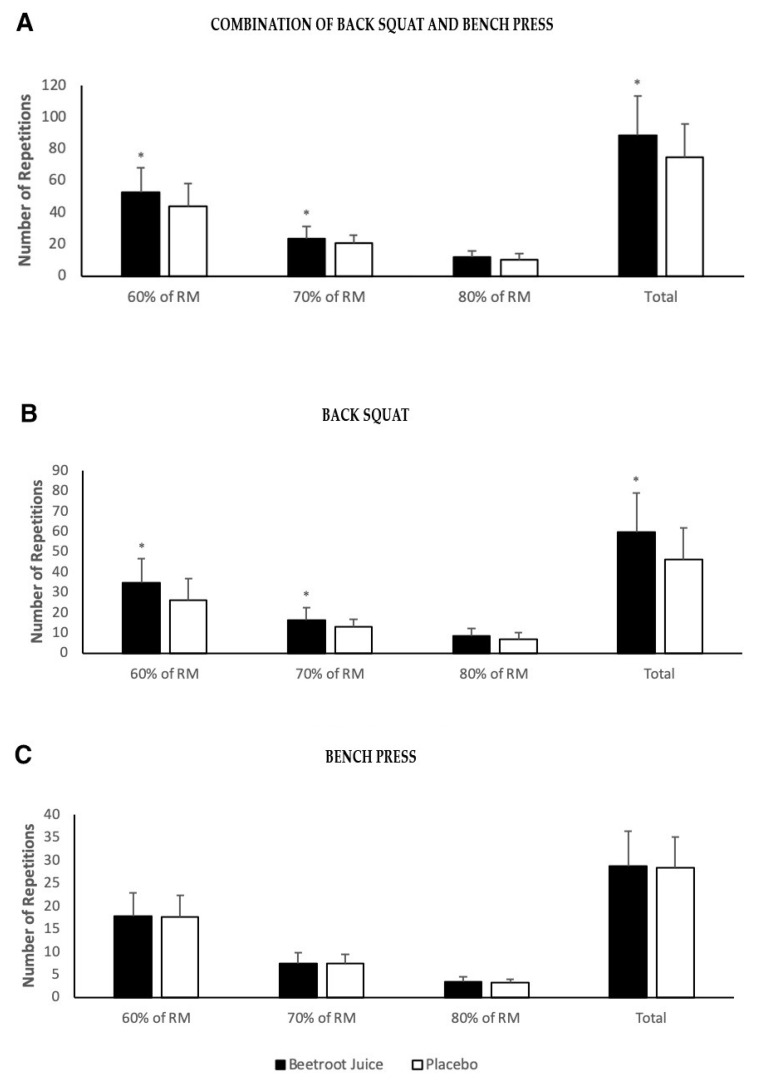
(**A**) Number of repetitions performed in the combination of back squat and bench press; (**B**) number of repetitions performed in back squat; (**C**) number of repetitions performed until concentric failure for each set of intensities 1-RM (60%, 70%, 80%) in bench press. RM: one-repetition maximum. (*) Significant difference between beetroot juice (BJ) and placebo (PLA) conditions (*p* < 0.05).

Significant effect for time (cumulative effect on series 60%, 70%, 80% 1-RM) and for supplement condition (BJ vs. PLA) was observed (*p* < 0.01), in addition to a time × condition interaction effect on total number of repetitions performed (*p* < 0.05). There was an increase in the total number of repetitions on the day BJ was consumed compared to the day the PLA was taken (MD: 13.8 ± 14.4; *p* < 0.01; ES = 0.6), in addition, according to the variable intensities in the exercise protocol, differences were found for 60% 1-RM (MD: 9 ± 10; *p* < 0.05; ES = 0.61), 70% 1-RM (MD: 3.1 ± 4.8; *p* < 0.05; ES = 0.49), but no differences were found for 80% 1-RM (MD: 1.7 ± 1; *p* = 0.12; ES = 0.41) (Figure 2A). 

Differences were found in the total repetitions of the back squat exercise (MD: 13.4 ± 13; *p* < 0.01; ES = 0.77), both at 60% 1-RM (MD: 8.7 ± 3.3; *p* < 0.01; ES = 0.78) and at 70% 1-RM (MD: 3.1 ± 2.5; *p* < 0.05; ES = 0.59), no differences were obtained at 80% 1-RM in favor of BJ (MD: 1.6 ± 0.9; *p* = 0.100; ES = 0.44) (Figure 2B). No differences were observed in the bench press exercise (MD: 0.4 ± 5.1; *p* = 0.785; ES = 0.06), for any percentage of intensity: 60% RM-1 (MD: 0.3 ± 3.4; *p* = 0.804; ES = 0.05); 70% 1-RM (MD: 0.1 ± 1.8; *p* = 0.878; ES = 0.04); 80% 1-RM (MD: 0.1 ± 1.3; *p* = 0.830; ES = 0.08) (Figure 2C).

For maximum velocity and maximum power, no differences were found after BJ or PLA consumption at the load of 60%, 70%, 80%, or total session in both exercises, back squat and bench press (*p* > 0.05) (Table 2). Significant effect for time (cumulative effect on series 60%, 70%, 80% 1-RM) was observed on velocity and maximum power (*p* < 0.01), but no effect for supplement condition (BJ vs. PLA) or time × condition interaction was observed (*p* > 0.05). 

Related to RPE after the sets, no differences were observed after BJ supplementation vs. PLA either in back squat (8.5 ± 1 vs. 8.2 ± 1.5; *p* = 0.41; ES = 0.23) or bench press (8.2 ± 1.2 vs. 8.4 ± 1.3; *p* = 0.48; ES = 0.16). Neither could we detect any differences in the lactate post-exercise measurement for BJ compared to that for PLA (12.85 ± 4.09 mmol·L^−1^ vs. 16.17 ± 6.17 mmol·L^−1^; *p* = 0.15; ES = 0.63).

## 4. Discussion

The aim of this study was to investigate the acute effects of BJ supplements compared to a PLA on the movement velocity and power produced in concentric phase during resistance exercise and on muscular endurance measures such as the maximal number of repetitions until failure. The results indicated that acute pre-training BJ supplements (120 min prior) improve muscular endurance in back squat exercise but not in bench press, without any effect on movement velocity or power production.

The recent Williams et al. study [15] was the first to measure the acute effects of BJ on power output and velocity during free-weight resistance exercise, specifically in bench press, in a population of resistance-trained men, finding improvements in mean velocity and power, in addition to a higher number of repetitions until failure in BJ compared to that in PLA, results that differ from those found in our study. The different results in velocity and power production with respect to Williams et al. [15] may be due to exercise equipment and the method used to measure the movement velocity, because our study was exclusively based on the analysis of the concentric movement phase of the exercise and was started from an isometric position, with a pause briefly after the eccentric phase (between 1 and 1.5 s), supported by either the bar or the chest, this reason could limit the differences in the mechanical variables associated with the load-velocity and the load–power profiles [31]. However, it would allow more reliable measurements in strength assessments based on movement velocity, in addition to the use of a Smith machine in order to limit the countermovement, enabling greater control and reproducibility of the measurement in the concentric phase of movement [31], unlike Williams et al. [15] who used a free-weight bench press in dynamic RT. In relation to this, other studies [38,39] demonstrated that BJ supplementation enhanced angular velocity and muscle power in knee extension evaluated in different populations, these results supporting the hypothesis that NO_3_^−^ influences the contractile properties of human muscle [38] according to previous studies in rats [17]. A previous study by Bender et al. [40] demonstrated that acute BJ supplementation increased peak isometric force output in adolescent men, although they used a higher dose and consumption time than ours: 2 × 70 mL of BJ (∼12.9 mmol·L^−1^ NO_3_^−^) at 2.5 h prior to performing test. In addition to this, Whitfield et al. [41] reported that BJ supplementation increased force production and peak twitch tension albeit using a multiday dosing protocol. 

The muscular endurance improvements found in our study, due to an increase in the number of repetitions performed coincided with previous studies [13,14,15]. Bailey et al. [13], showed that a supplementation period of 6 days with BJ improves knee extension exercise challenges over multiple sets, reducing the total use of ATP and increasing NO_3_^−^ concentrations in plasma. Similarly, over a six day period, Mosher et al. [14], have demonstrated, in a study with 12 active men comparing BJ supplementation versus PLA, how strength performance was increased with BJ supplementation in terms of weight lifted and the number of repetitions. Bolstering this, previous evidence may suggest that longer loading may be needed to maximize benefits of BJ supplementation in resistance-trained men, however, the participants in the present study took supplements only once, 120 min before the strength training session, with the aim of evaluating a single dose (6.4 mmol·L^−1^ or 400 mg NO_3_^−^) prior to BJ training, and obtained an improvement in muscular endurance during RT comparable to that found in the Williams et al. study [15]. However, another study [18], with an exercise protocol similar to ours, designed to assess neuromuscular fatigue, with loads initially set at 60% 1-RM and increasing by 10% 1-RM per set to 90%, before descending by the same increments back to 60% 1-RM, did not find improved performance on RT after 3 days supplementation with an NO_3_^−^ rich supplement bar (∼1.1 mmol·L^−1^ or 35.2 mg NO_3_^−^). There was the possibility of not finding improvement in the RT performance through this investigation, because the quantity could be insufficient, being less than that of Mosher et al. [14], Williams et al. [15], and that of our work and even, to what is recommended in other investigations [1] (∼6.4 mmol·L^−1^ or 400 mg NO_3_^−^). However, in this study [18], an improvement in the ability to sustain the peak EMG amplitude was observed, which might suggest that an NO_3_^−^ rich supplement could enhance neuromuscular efficiency during fatiguing resistance exercise. Future studies should seek to elucidate optimal supplement dosage for RT performance, since it seems that an acute pre-exercise consumption could improve performance in resistance-trained men, thus avoiding a longer consumption. 

In our study, the results on bench press did not coincide with the increase in the number of repetitions shown by Williams et al. [15], but there was a significant improvement in back squat (exercise performed before the bench press) and the total of the session (back squat and bench press), exercise that suggesting that the authors should explore [15], in addition to different relative intensities, measurements similar to those in our study, i.e., 60%, 70%, 80% 1-RM. These differences between upper-body and lower-body could be due to a limited ability to increase the recruitment of motor units in smaller muscles (lower body) compared to recruiting those in large muscles, as previously observed in similar conditions of testing with the intake of other supplements such as caffeine [42], in addition to the vasodilatory action of the BJ [43] and the fact that back squat is a strength exercise with greater involvement of several muscle groups and which involve a greater muscle volume [44], requiring a great deal of cardiovascular effort [2]. We would like to stress that the bench press exercise was the second test performed in the assessment procedure, and the performance of the participants, therefore, may have been affected by the accumulated neuromuscular fatigue, which could also be caused by the accumulation of metabolic byproducts [45]. 

Improvement results in muscular endurance during RT could be based on the role of NO, as it participates in blood pressure regulation [46] thanks to its vasodilatory effect, along with its ability to inhibit blood coagulation [47]. In addition to endogenous production from the oxidation of L-arginine, there is a metabolic oxidation route, NO_3_^−^–NO_2_–NO, which is independent of NO synthase [48]. Contributing NO_3_^−^ via BJ therefore seems a more practical way to improve the NO production, favoring a greater volume of blood, and hence oxygen, in sports involving an important muscular endurance component. This is even more important when the sports characteristically lead to an acidic and hypoxic body environment, a situation where the metabolic reduction of NO_3_^−^ increases the activity of the NO precursor supplement [48]. A greater oxygen supply delays the onset of muscle fatigue in sports performance [49] and leads to increased energy production from ATP via aerobic metabolic pathways, delaying the onset of anaerobic metabolic pathways, since more intense and longer-lasting physical activity leads to higher oxygen consumption (VO_2_) in order to generate the required ATP [50]. As a result, the use of NO_3_^−^ could increase sports performance, since its vasodilatory function and the greater supply of oxygen to the muscles increases the subjects’ maximum VO_2_ (VO_2max_) and reduces the oxygen consumption for the exercise. All this would lead to a decrease in the ATP cost and thus mitigate the changes in intramuscular substrates and metabolic production (PCr, ADP, Pi), increasing muscle oxygenation [51], delaying the onset of muscle fatigue. Consequently, reduced oxygen consumption and a lower ATP cost would delay lactate production [49]. In this way, since lactate is an indicator of the contribution of glycolysis to metabolism [52], having obtained an increase in the number of total repetitions after the consumption of BJ with the same concentrations of blood lactate could suggest an energy efficiency. Accordingly, Bailey et al. [13] demonstrated that BJ consumption reduces both the O_2_ costs and the degree of PCr degradation during both low- and high-intensity exercise without affecting muscle pH, improving exercise efficiency, and this could explain the greater load carried by the subjects when they consumed BJ [13]. Although in our study the same blood lactate values were maintained, another study observed a lower value after the consumption of BJ compared to the PLA, during RT [14]. 

Another physiological basis for current results may be due to the type-specific effects of fiber in NO_3_^−^ supplementation [53]. Ferguson et al. [8] demonstrated that BJ supplementation increased limb blood flow and skeletal muscle in preference to fast-twitch fibers (type II). Since O_2_ supply is a limiting factor in ATP-PC resynthesis and lactate clearance may affect muscle strength production [10,54], improved blood flow to type II fibers may have resulted in improved and sustained muscle strength production leading to improved resistance exercise performance. Although our study did not aim to explore exercises with different proportions of muscle fibers, it could be an interesting line of research for the future.

Several studies have assessed the effect of BJ supplementation on RPE. Although some authors have reported improved performance without any significant impact on RPE [12,14,55], others have shown lower RPE values [56,57,58]. To the authors’ knowledge, there is only one study that recorded the effect of BJ on RPE during RT [14]; Mosher et al. [14] found that RPE during muscular endurance in RT was not significantly affected by 6 days of dietary nitrate supplementation. These results were in agreement with those of the present study, which measured the acute effects of BJ. However, both in Mosher’s study [14] and in ours, it should be noted, that there was an improvement in performance with BJ consumption without causing any change in RPE, therefore, BJ might increase tolerance during RT maintaining the same RPE. Among the possible mechanisms that could explain the effects of BJ on RPE is an improvement in blood flow to the frontal lobe of the brain which regulates decision-making and motor control, contributing to subjective perception of effort [59], being able to cause a possible increase in exercise performance [57]. In addition to this, it should be noted that maintaining RPE after BJ supplementation could be the result of reduced central motor command as a result of preserved contractile function during exercise [58], since it has been claimed that RPE reflects a mechanism of centrally mediated feedback in which a copy of the central motor command output is sent from the motor areas to the sensory brain to allow conscious awareness of the processes associated with motor output [60]. During fatigued contractions, the progressive increase in RPE could reflect the increase in central motor command, necessary to compensate for exercise-induced deficiencies in contractile muscle function to ensure adequate power output to maintain the task [61].

Although this study collected information about the identification of BJ, its main objective was not to study how the possible identification could affect the results or not, as has been done previously with other supplements such as caffeine [62] or creatine [63] in sports performance. We therefore suggest investigating the placebo effect of BJ on sports performance as a future line of research.

## 5. Limitations

Our study has several limitations. Although, it is known that a dosage of BJ similar to that in this study significantly increases plasma NO_2_^−^ [1,16], plasma NO_2_^−^ was not measured before the intake by the participants of BJ or PLA groups, leaving the concentration needed to accompany resistance exercise improvements unknown. 

Furthermore, the incremental test protocol (60%, 70%, 80% 1-RM) used in our study, could generate a possible accumulated fatigue in the subsequent series. Our study only investigated a range of relative intensity of moderate loads, therefore, the ergogenic effect on neuromuscular performance of BJ could be investigated in a wider range of relative loads, used with different orientation, as has been studied in supplements rich in NO_3_^−^ or other supplements [18,64].

## 6. Conclusions

In conclusion, the acute ingestion of a BJ supplement 120 min before training seems produced an ergogenic effect on the muscular endurance performance in RT, particularly, increasing total repetitions performed, accompanied by the same RPE and without changes in blood lactate levels in participants. However, BJ supplementation did not improve performance in terms of movement concentric velocity and power in back squat and bench press. These findings suggest that BJ supplementation could be a suitable strategy to improve muscular endurance, increasing tolerance to RT.

## Figures and Tables

**Table 1:** Descriptive characteristics and anthropometric data of participants.

Variable	Value
AGE (YEARS)	24 ± 3
HEIGHT (M)	1.75 ± 0.08
WEIGHT (KG)	73 ± 9.2
BODY MASS INDEX (KG/M2)	23.75 ± 2.6
BODY FAT (%)	15.8 ± 4
MANUAL PRESSURE FORCE (KG)	47.8 ± 7.1
RT EXPERIENCE (YEARS)	3.2 ± 0.9
1-RM BACK SQUAT (KG)	93.2 ± 18.4
1-RM BENCH PRESS (KG)	80.3 ± 19.2

Data expressed as mean ± standard mean. 1-RM: one-repetition maximum; RT: resistance training.

**Table 2:** Movement concentric velocity control for back squat, bench press, and total session after the consumption of placebo or beetroot juice.

Variables	Back Squat				Bench Press				Total Session			
	PLA	BJ	*p*-Value	ES	PLA	BJ	*p*-Value	ES	PLA	BJ	*p*-Value	ES
**Maximum Velocity (m·s^−1^)**												
60% 1-RM	0.685 ± 0.094	0.697 ± 0.090	0.590	0.13	0.612 ± 0.082	0.609 ± 0.080	0.894	0.04	0.526 ± 0.051	0.522 ± 0.054	0.715	0.07
70% 1-RM	0.611 ± 0.076	0.605 ± 0.083	0.727	0.07	0.431 ± 0.083	0.433 ± 0.058	0.896	0.03				
80% 1-RM	0.512 ± 0.056	0.513 ± 0.085	0.971	0.01	0.306 ± 0.052	0.275 ± 0.053	0.135	0.59				
Average	0.603 ± 0.067	0.605 ± 0.075	0.877	0.02	0.449 ± 0.059	0.440 ± 0.051	0.414	0.16				
**Maximum Power (Watt)**												
60% 1-RM	382 ± 111	389 ± 117	0.538	0.06	292 ± 94	289 ± 88	0.777	0.03	320 ± 90	318 ± 86	0.599	0.03
70% 1-RM	395 ± 107	393 ± 116	0.816	0.02	238 ± 81	242 ± 81	0.690	0.05				
80% 1-RM	378 ± 96	377 ± 108	0.961	0.01	191 ± 55	176 ± 66	0.257	0.25				
Average	392 ± 112	390 ± 104	0.857	0.02	249 ± 76	245 ± 77	0.520	0.05				

BJ: beetroot juice; m·s^−1^: meters/seconds; ES: effect size; PLA: placebo; 1-RM: one-repetition maximum. Significance was analyzed according to paired-samples t-test. ES was calculated following the method set out by Lakens [36] using Cohen’s *d* as the ES index, where ES could be roughly classified as small (<0.2), medium (0.5 to 0.8), and large (>0.8) [37]. Note: data expressed as mean ± standard deviation.

## Supplementary Materials

The following are available online at https://www.mdpi.com/2072-6643/12/7/1912/s1, Table S1: Post-exercise BJ supplement identification responses.
