# Supplementary material for: Acute Effects of Beetroot Juice Supplements on Resistance Training: A Randomized Double-Blind Crossover
Source: Nutrients. 2020 Jun 28;12(7):1912. doi: 10.3390/nu12071912 (PMC7401280; doi:10.3390/nu12071912)
Supplement: Supplementary file 1 [file nutrients-12-01912-s001.pdf]

Supplementary Table S1. Post-exercise BJ supplement identification responses

	**Correct**	**Don't know**	**Wrong**
N*	8	2	2
BJ IDENTIFICATION (%)*	66%		

BJ: beetroot juice. *The answers presented are shown for BJ and not for PLA, because if the participants changed their answer on the second test day, this last answer was taken into account.
